# Transactions of the Chicago Gynæcological Society

**Published:** 1879-11

**Authors:** W. H. Byford


### Society Reports.

### ARTICLE XI.

TRANSACTIONS OF THE CHICAGO GYNÆCOLOGICAL SOCIETY. NINTH MEETING, SEPTEMBER 26TH, 1879. W. H. BYFORD, M.D., in the chair.

DR. MERRIMAN read a paper on Perimetric Inflammations (published in the present number of the JOURNAL AND EXAMINER.)

DISCUSSION.

DR. NELSON:—I would call attention to the importance of a correct diagnosis, for if the attention of the physician is directed simply to the metritis or endometritis, and a treatment adopted which would be appropriate were these the only diseases present, *with no perimetritis*, the patient is likely to be made worse, rather than better, should perimetritis also, in a chronic form, be present; as the applications will then be too strong. We should always first ascertain the mobility of the uterus before making any application. If its movements are restricted in any direction, it is usually from the effects of perimetritis; and if perimetritis has produced this sluggish mobility of the uterus, it is almost certain that strong applications to the interior will arouse the inflammation, latent in a chronic form, and transform it into an acute inflammation with its attendant dangers. When perimetritis exists, any application is too strong which produces severe pain for an hour or more.

If such pain is not produced, carbolic acid, a solution of iodine, and even the full strength pure nitric acid may be applied to the os, at another time to the cervix, and finally to the whole uterine cavity with safety and advantage, provided the proper care be exercised as to the nearness of menstruation. But if much lasting pain is produced, even the introduction of the sound may be a dangerous operation, and much more so any application to the interior of the uterus.

I had the opportunity to-day of examining a pregnant uterus in a patient having varicose veins of the left limb, extending even to the vulva. I was much interested to find the left side of the cervix, and so much of the uterus as could be examined, had the bluish appearance and the peculiar feel of a varix, while the right side was nearly or quite normal.

If we remember the difficulty with which ulcers, resulting from slight abrasions, heal upon the leg, when the veins are in a varicose condition, I think we shall in part understand why inflammation around the uterus is so likely to become chronic and then so difficult to completely remove. The normal genital veins have many points of resemblance to a varix.

In seeking for the causes of perimetritis, I would emphasize traumatism—the injury of the tissues about the uterus by violence, by downward pressure, as in falls, lifting and the like; and by upward pressure, as in proper coitus, especially where there is a great disproportion between the male and female organs, particularly if there be added the disturbance of the nervous system in a "cold." Hence, we find in hospitals that prostitutes are frequently sufferers from perimetritis, both acute and chronic.

Finally, a caution in the use of hot water, which is a valuable therapeutic agent in both the acute and chronic forms of perimetritis, if properly used.

In the acute form, the interrupted, forcible stream should rarely be used, and never if it produces continuing pain; while in the chronic form, the intermediate stream is perhaps preferable, but the force should not be sufficient to produce lasting pain.

In answer to a question, Dr. Nelson said he thought in those cases where injury was inflicted during coitus, it was due to the stretching of the ligaments and attachments of the uterus by the disproportionate male organ.

DR. MILLER said the subject introduced by Dr. Merriman for this evening's conversation was one of great interest. Perimetritis was a disease of frequent occurrence. The consequences liable to follow it were many times serious, and not infrequently disastrous. The varied terms used to designate this inflammation was evidence of an attempt at over-refinement by writers. He doubted whether inflammation, especially if acute, was limited to a single structure within the female pelvis. Perimetritis is a term sufficiently definite for our present purpose.

The causes of perimetritis are exceedingly numerous. Mechanical injury is doubtless a common cause. How it is possible for the delicate tissues within the pelvis to escape, during parturition, may well excite our wonder. A hard body (the fœtal head) is forced through an unyielding canal (the pelvis) with such violence, that it would seem every tissue would be contused and lacerated beyond the possibility of recovery. To these contingencies and to others of a mechanical nature, we may add as frequent exciting causes, exposures to cold, especially during menstruation. These exposures occur in ways too numerous to mention. A vicious habit, which he believed to be very prevalent, and should be strongly condemned, was a resort to cold vaginal injections, immediately after coitus. If Noegerath's startling theory, of the baleful influence of latent gonorrhœa, be well founded, we should not be surprised that perimetritis is common, for it is easy to understand how specific inflammation may extend to the interior of the uterus, the Fallopian tubes, ovaries and surrounding tissues. Perimetritis sometimes unexpectedly follows, as an accident, slight operations within the pelvis.

It is not usually difficult to diagnose a case of perimetritis, and yet it is no uncommon circumstance, that symptoms due to perimetritis are for a long time treated as diseases, which in the given cases do not exist. Some of the most frequent errors arise where the inflammation involves tissues contagious to the origin of the sciatic nerve, leading the unwary to suppose that the remedies for sciatica are indicated; or the bladder or the rectum may become similarly involved.

The history of the case, the pulse, the temperature and a careful exploration of the pelvis, would usually obviate these errors.

He agreed in the main with the suggestions made regarding the treatment and would not repeat them. He would, however, add as an important measure, counter-irritation. A blister of large size applied over the hypogastrium, to be followed by fomentations for a few hours, then the following to be substituted:

| | | |
|---|---|---|
| ℞ Ung. hydrarg | 16 | grams. |
| Pulv. camph | 4 | " |
| Pulv. opii | 2 | " |

M.

The hot injections to be followed by carbolized cotton saturated with glycerine, to which a solution of opium should be added.

Should suppuration take place, and fluctuation be distinct, an opening into the abscess would be judicious.

DR. ROLER thought it unfortunate that so many confusing terms were employed to designate inflammations about the uterus. The distinctions between perimetritis, parametritis, etc., as expressed by some gynæcologists, cannot always be made, as it seldom happens that one set of tissues are singly affected.

Pelvic peritonitis generally involves more or less of the connective tissue beneath, and in cases where cellulitis with effusion becomes the important feature, the contiguous parts or organs are apt to be implicated either primarily or secondarily. In his opinion, acute perimetritis, as defined by the essayist, seldom occurs as a primary affection, but is an extension from an inflamed uterus or ovaries brought about by some aggravating circumstance.

The treatment of acute forms of pelvic inflammation should be decidedly antiphlogistic. Leeches, followed by hot fomentations over hypogastrium and sitz-baths, are the chief local measures. Opiates should be given freely. In the chronic forms, blistering, as recommended by Dr. Miller, and hot water injections in the vagina, were of greatest service.

DR. SAWYER had also noticed the fact to which attention had been drawn by Dr. Nelson, that prostitutes were often subjects of perimetric inflammations, and had attempted to find an explanation of this frequency. He could not accept the view of that gentleman that it was due to violent stretching of the uterine attachments, for the latter are purposely and often very rudely stretched by the surgeon, during certain operations upon the organ, and yet inflammations of the surrounding parts almost never result as a consequence. Nor could the speaker fully accept the view advanced by others, that the male organ could so bruise the female parts as to excite perimetric inflammations. When it is recalled that these parts are subjected to severe contusion by the passage of a snugly fitting fœtal head during parturition, usually without this consequence; the speaker thought the bruising incident to a most violent coitus must be much less.

After giving some thought to this subject, Dr. Sawyer said, he believed the frequency of perimetric inflammations in abandoned women, and perhaps in some others also, found its explanation in the pernicious and disgusting habit of permitting coitus during or very near the period of menstruation. The effect of this obviously being to increase the natural hyperæmia to a dangerous degree.

He had also observed that some women after abortion seemed liable to perimetric inflammations, which, he was convinced, had the same explanation; unseasonable or immoderate coitus not only exciting the abortion, but inducing a degree of congestion which made the woman an easy victim to perimetric inflammations afterward.

DR. EARLE said that he would call the attention of the Society to simply one cause of perimetritis, namely, the use of those instruments and appliances for dilating or medicating the cervical canal:

1st. The use of slippery elm, sponge or laminaria tents; and

2d. The use of various metallic dilating instruments.

In supporting his proposition, he said that while in many cases great good had seemed to follow this line of treatment, many cases of dysmenorrhœa and sterility being relieved and cured, he thought that great care should be exercised in their use.

He related a case in practice where a very small slippery elm tent was introduced — the utmost care being used — but a very severe inflammation, followed by abscess, was the result.

A second, recent, case was also related. A lady, sterile for years, was being treated by the use of a series of metallic bougies. And although the introduction of these instruments had been made at intervals of several days, and the dilatation carried on very slowly, at last a slight perimetritis was produced.

DR. JONES alluded to the comparative anatomical position of the right and left utero-sacral ligaments as favoring the occurrence of inflammation on the left side of the pelvis, rather than the right. He thought the use of sponge tents prolific of cellulitis, and mentioned, as a frequent cause, the abstraction of heat from the soles of the feet by the cold iron of sewing-machine treadles, especially during menstruation.

As a matter of interest in prognosis, it was important to determine, if possible, whether inflammation pervaded mainly serous tissues or the areolar — the latter being far more prone to suppuration.

